# Supplementary material for: A genome-wide survey of mutations in the Jurkat cell line
Source: BMC Genomics. 2018 May 8;19:334. doi: 10.1186/s12864-018-4718-6 (PMC5941560; doi:10.1186/s12864-018-4718-6)
Supplement: Supplementary file 1 — Supplementary Materials. Supplementary figures S1 to S8 and captions for supplementary tables S1 and S2. (PDF 2361 kb) [file 12864_2018_4718_MOESM1_ESM.pdf]

Supplementary Materials for

A Genome-wide Survey of Mutations in the Jurkat Cell Line

Louis Gioia*, Azeeem Siddique, Steven R. Head, Daniel R. Salomon, & Andrew I. Su

*To whom correspondence should be addressed; E-mail: lhgioia@scripps.edu

This PDF file includes:

Figs. S1 to S8
Captions for Tables S1 and S2

Figure S1: Raw CNVnator read depth density
Distribution of raw CNVnator read depth calls.

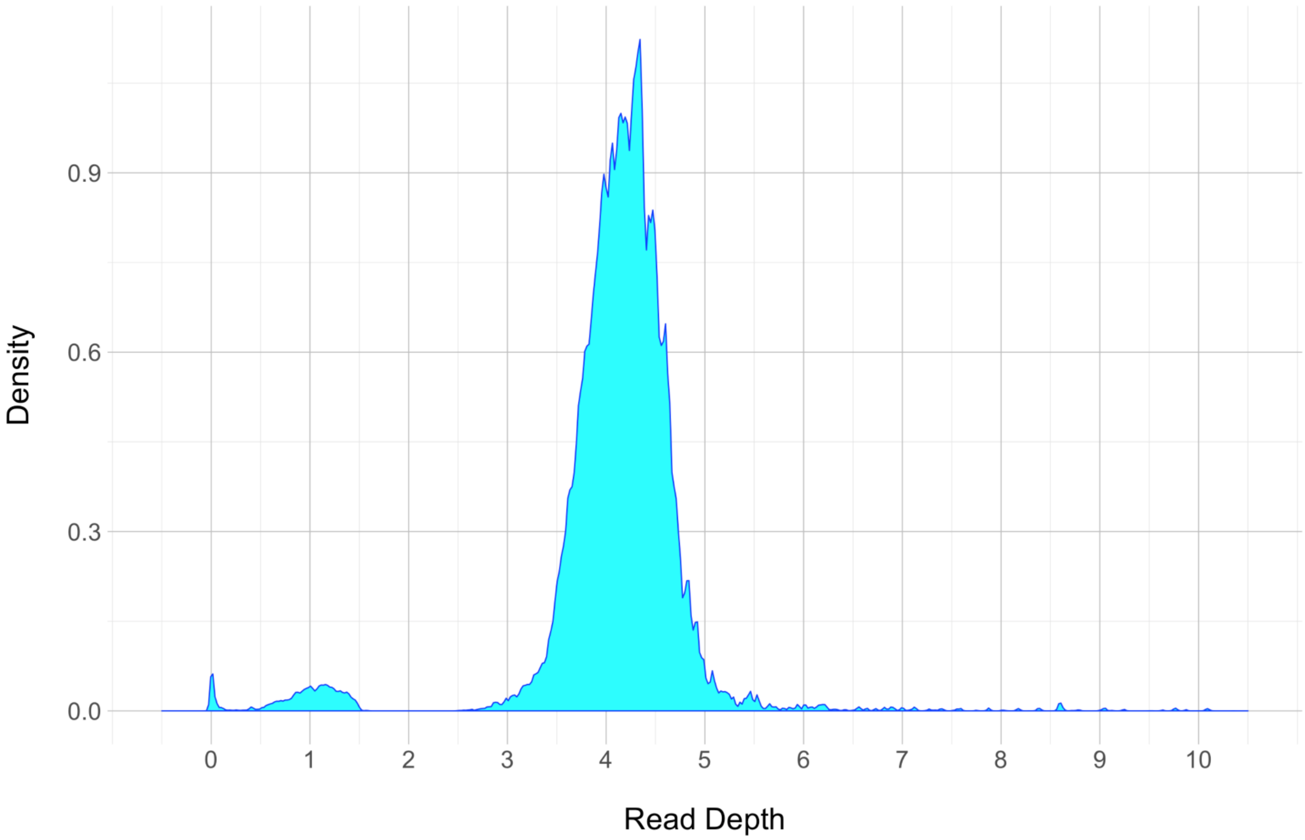

Figure S2: Deletion size distributions by variant caller (1 – 200bp)
Distributions of deletion call sizes between 1bp and 200bp for each variant caller. The bin size is 1bp.

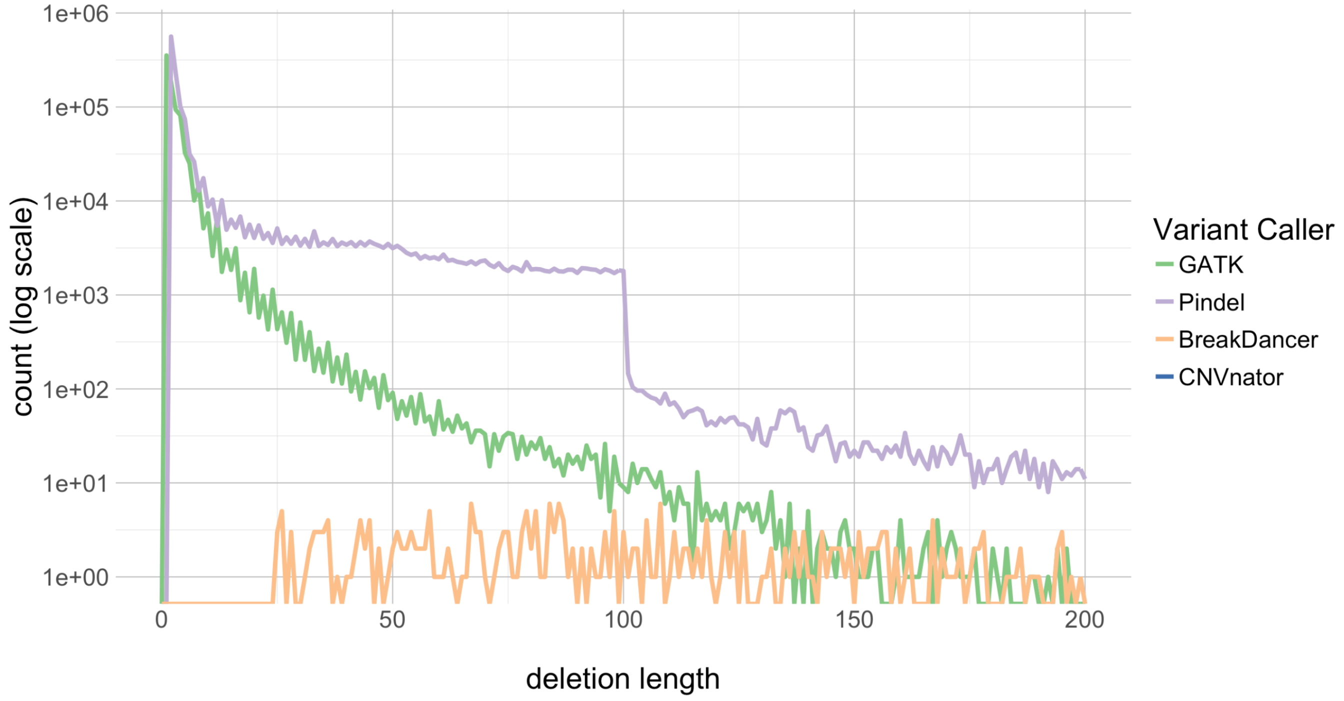

Figure S3: Deletion size distributions by variant caller (200 – 1,000bp)
Distributions of deletion call sizes between 200bp and 1kb for each variant caller. The bin size is 10bp.

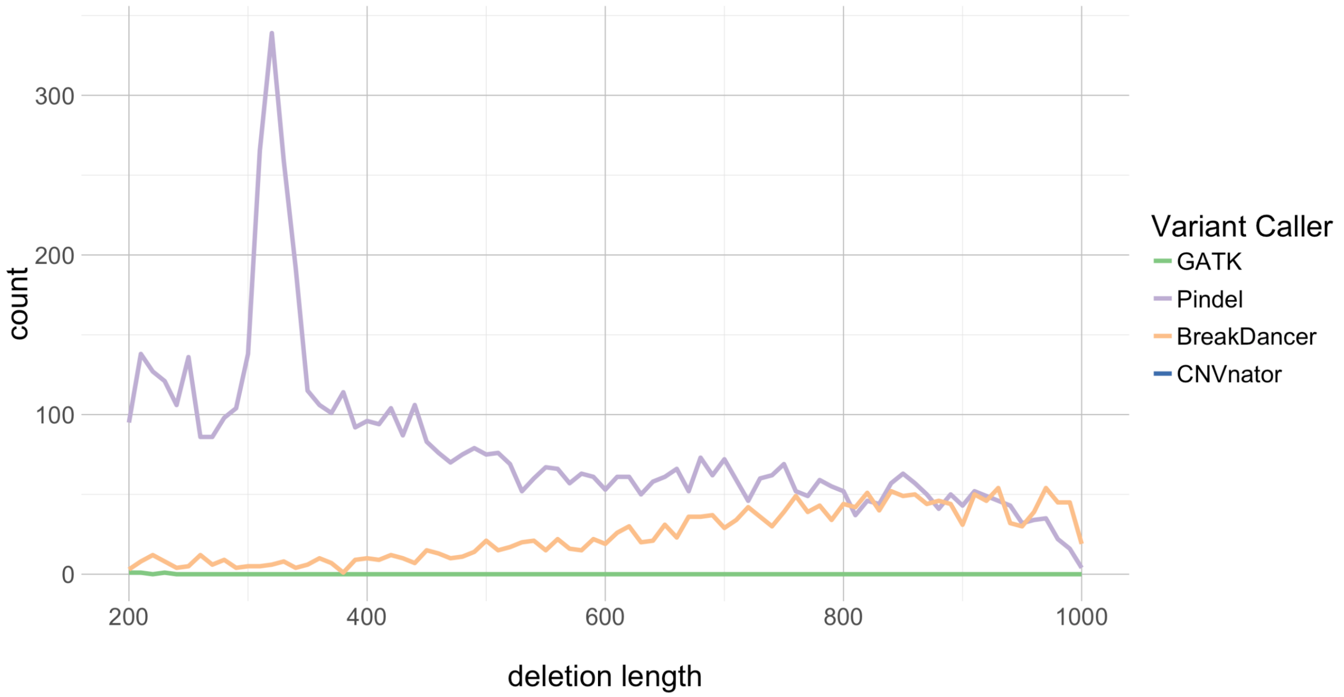

Figure S4: Deletion size distributions by variant caller (1,000 – 100,000,000bp)
Distributions of deletion call sizes between 1kb and 100Mb for each variant caller. The bin size is log-scaled with 200 bins.

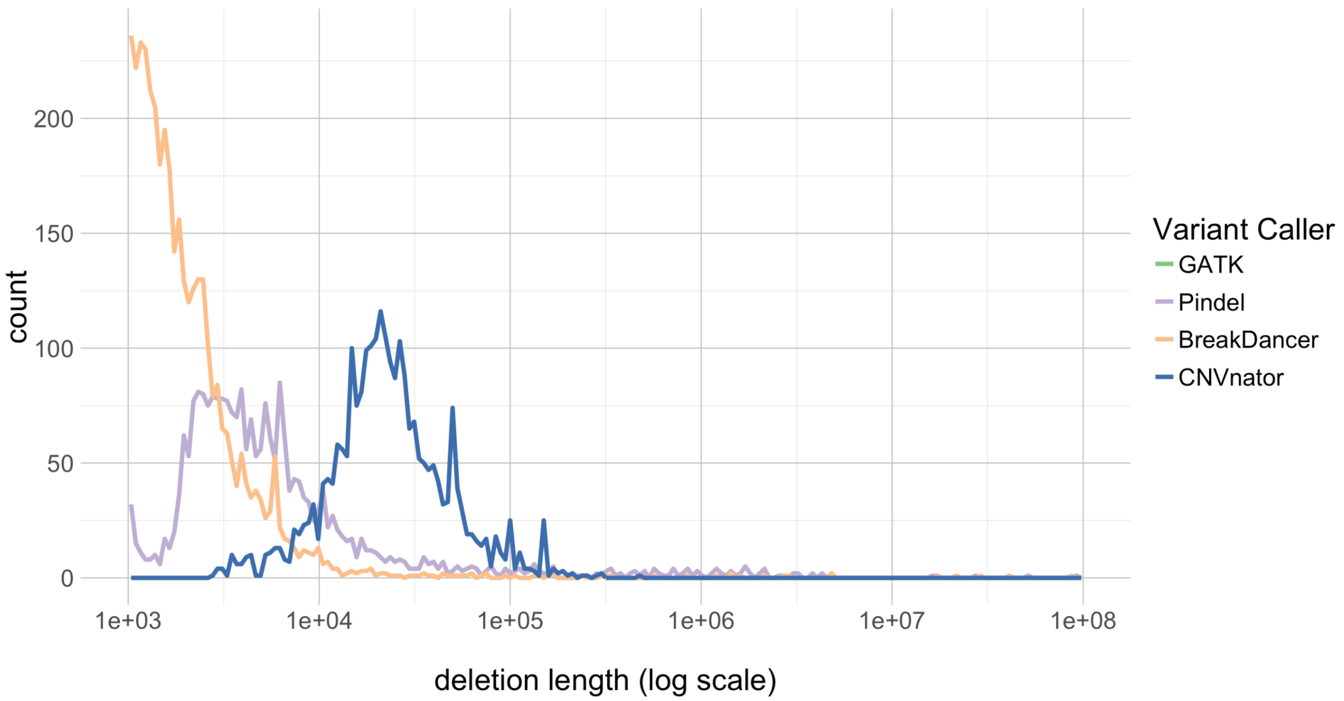

Figure S5: Insertion size distributions by variant caller
Distributions of insertion call sizes for each variant caller. The bin size is 1bp.

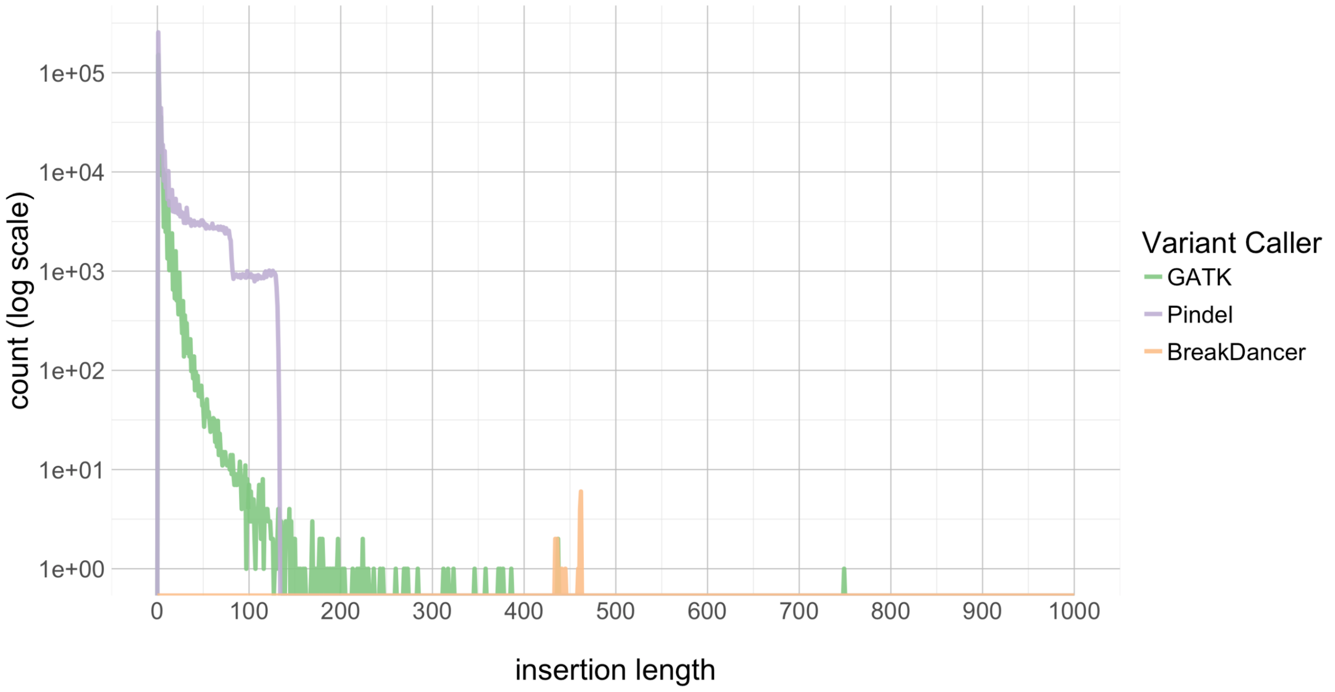

Figure S6: Duplication size distributions by variant caller
Distributions of duplication call sizes for each variant caller. The bin size is log-scaled with 200 bins.

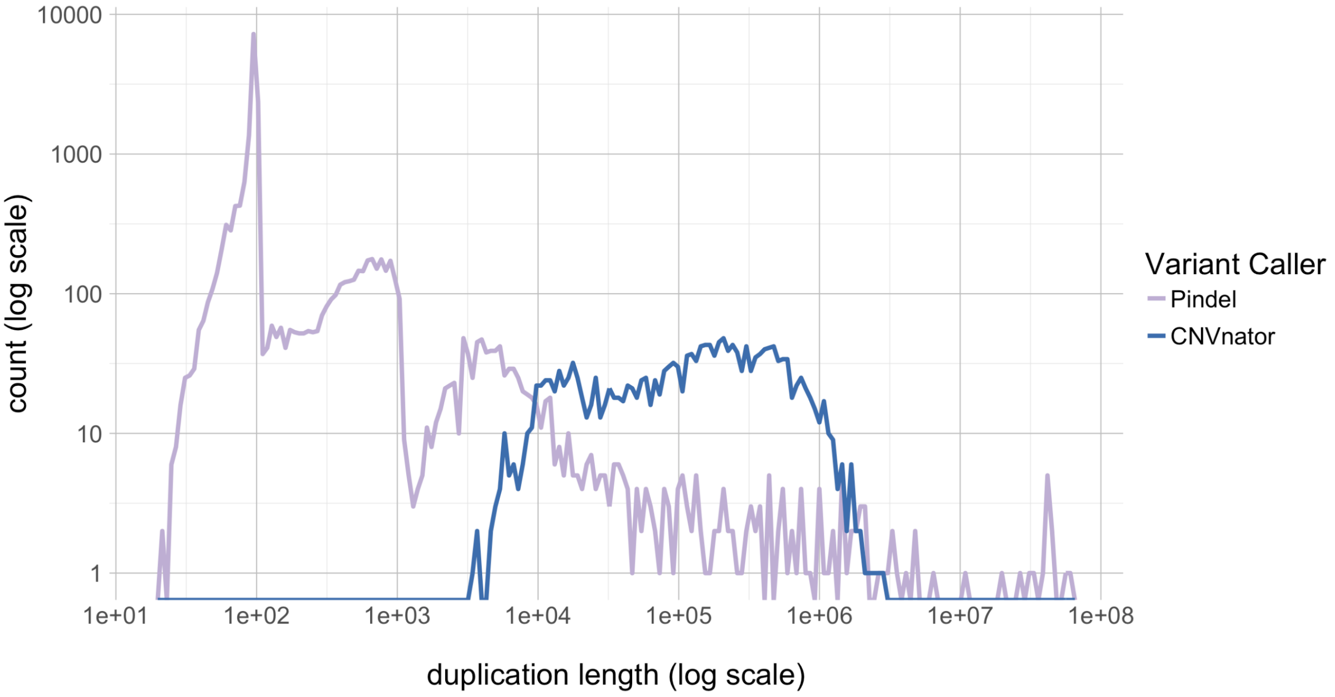

Figure S7: Inversion size distributions by variant caller
Distributions of inversion call sizes for each variant caller. The bin size is log-scaled with 100 bins.

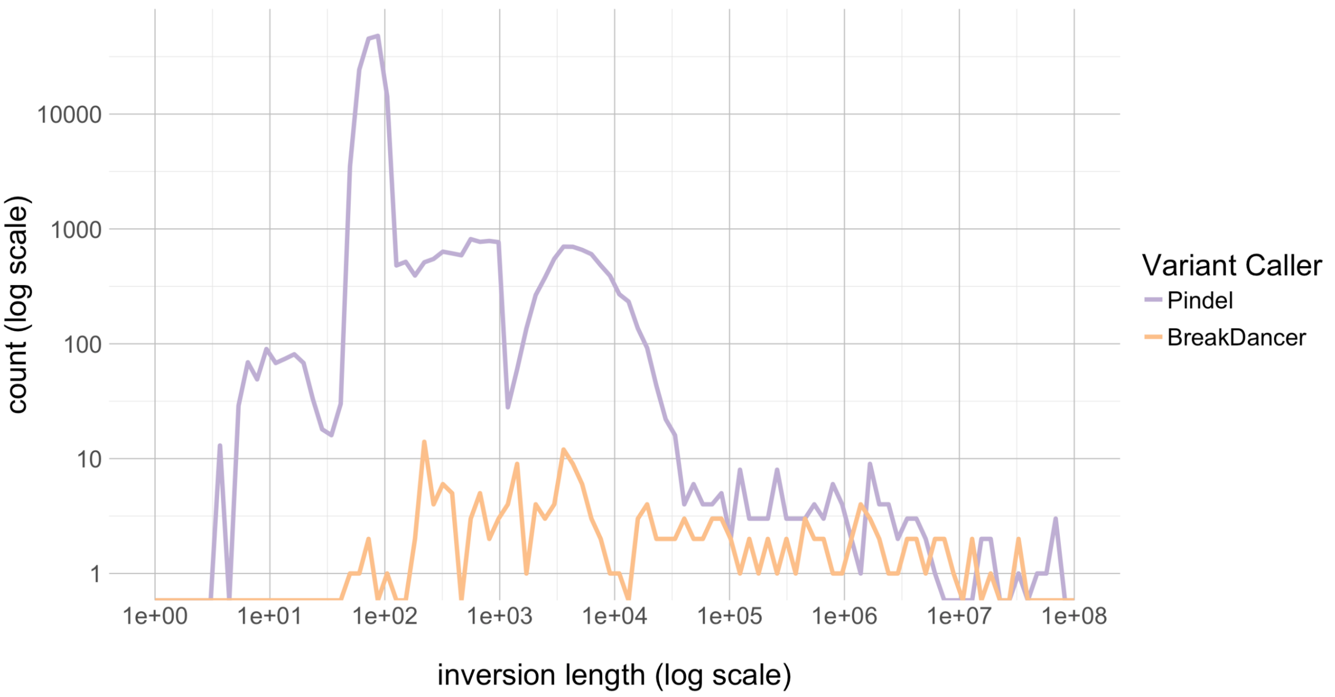

Figure S8: Intra-chromosomal translocation size distributions by variant caller
Distributions of intra-chromosomal translocation call sizes for each variant caller. The bin size is log-scaled with 100 bins.

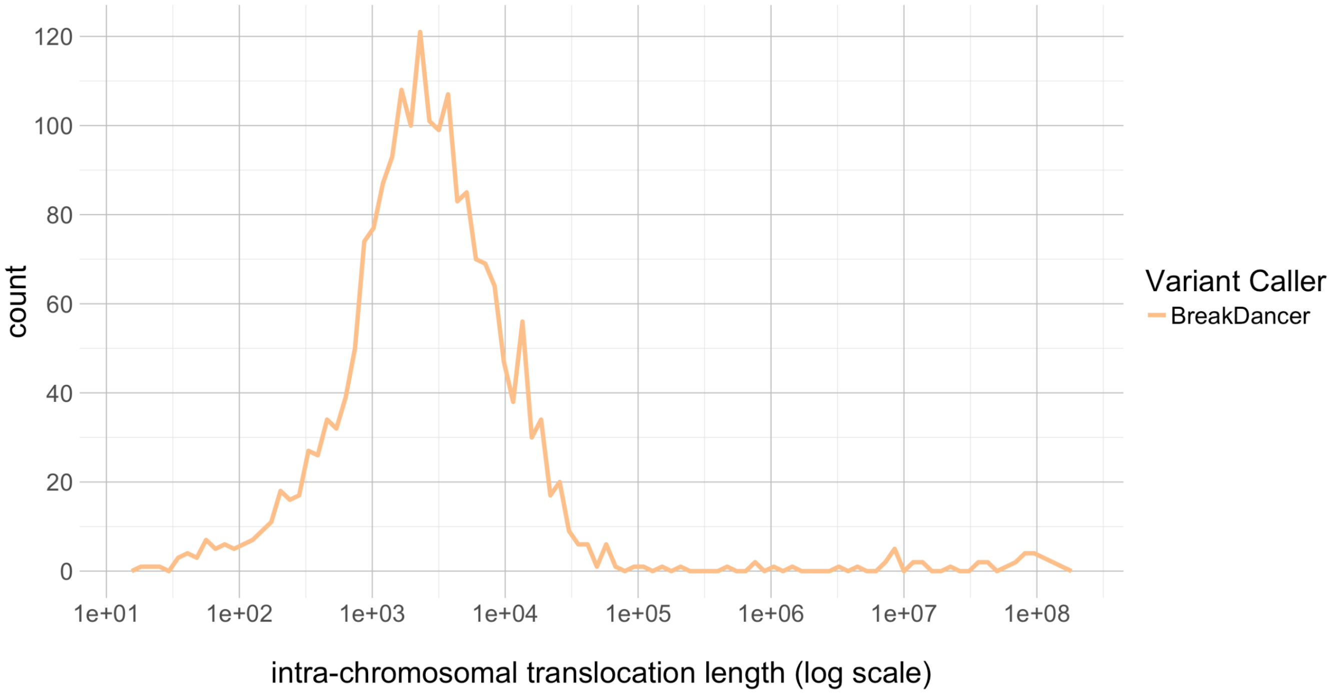

Table S1: Long deletions found in Jurkat with matches to pathogenic variants in dbVar
Matches were determined by 90% reciprocal overlap. All database matches are included with one dbVar entry per row.

Table S2: Duplications found in Jurkat with matches to pathogenic variants in dbVar
Matches were determined by 90% reciprocal overlap. All database matches are included with one dbVar entry per row.
